# Genetically predicted 486 blood metabolites in relation to risk of colorectal cancer: A Mendelian randomization study

**DOI:** 10.1002/cam4.6022

**Published:** 2023-05-03

**Authors:** Zhangjun Yun, Ziwei Guo, Xiao Li, Yang Shen, Mengdie Nan, Qing Dong, Li Hou

**Affiliations:** ^1^ Dongzhimen Hospital Beijing University of Chinese Medicine (BUCM) Beijing China; ^2^ Department of Oncology and Hematology, Dongzhimen Hospital Beijing University of Chinese Medicine (BUCM) Beijing China

**Keywords:** blood metabolites, causality, colocalization analysis, colorectal cancer, Mendelian randomization

## Abstract

**Background:**

Metabolic disorders are a hallmark feature of cancer. However, the evidence for the causality of circulating metabolites to promote or prevent colorectal cancer (CRC) is still lacking. We performed a two‐sample Mendelian randomization (MR) analysis to assess the causality from genetically proxied 486 blood metabolites to CRC.

**Methods:**

Genome‐wide association study (GWAS) data for exposures were extracted from 7824 Europeans GWAS on metabolite levels. GWAS data for CRC from the GWAS catalog database GCST012879 were used for the preliminary analysis. The random inverse variance weighted (IVW) is the primary analysis for causality analysis while MR‐Egger and weighted median as complementary analyses. Cochran Q test, MR‐Egger intercept test, MR‐PRESSO, Radial MR, and leave‐one‐out analysis were used for sensitivity analyses. For significant associations, additional independent CRC GWAS data GCST012880 were used for replication analysis and meta‐analysis. For the final identification of metabolites, Steiger test, linkage disequilibrium score regression, and colocalization analysis were performed for further evaluation. Multivariable MR was performed to assess the direct effect of metabolites on CRC.

**Results:**

The results of this study indicated significant associations between six metabolites pyruvate (odds ratio [OR]: 0.49, 95% confidence interval [CI]: 0.32–0.77, *p* = 0.002), 1,6‐anhydroglucose (OR: 1.33, 95% CI: 1.11–1.59, *p* = 0.002), nonadecanoate (19:0) (OR: 0.40, 95% C I:0.4–0.68, *p* = 0.0008), 1‐linoleoylglycerophosphoethanolamine (OR: 0.47, 95% CI: 0.30–0.75, *p* = 0.001), 2‐hydroxystearate (OR: 0.39, 95% CI: 0.23–0.67, *p* = 0.0007), gamma‐glutamylthreonine (OR: 2.14, 95% CI: 1.02–4.50, *p* = 0.040) and CRC. MVMR analysis revealed that genetically predicted pyruvate, 1‐linoleoylglycerophosphoethanolamine and gamma‐glutamylthreonine can directly influence CRC independently of other metabolites.

**Conclusion:**

The current work provides evidence to support the causality of the six circulating metabolites on CRC and a new perspective on the exploration of the biological mechanisms of CRC by combining genomics and metabolomics. These findings contribute to the screening, prevention and treatment of CRC.

## Graphical abstract

Due to unavoidable study design weaknesses, previous studies have made it difficult to assess the causality from circulating metabolites to colorectal cancer. In this work, the authors used the most comprehensive blood metabolite genome‐wide association study data to assess the causality between metabolites and colorectal cancer based on a Mendelian randomization study. They found that genetic liability for higher levels of pyruvate, nonadecanoate (19:0), 1‐linoleoylglycerophosphoethanolamine, and 2‐hydroxystearate associated with lower colorectal cancer risk while higher levels of 1,6‐anhydroglucose and gamma‐glutamylthreonine increased risk of colorectal cancer.

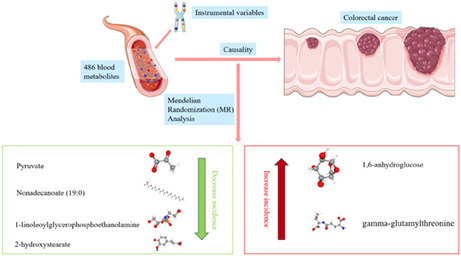

## 1 INTRODUCTION

Colorectal cancer (CRC) is currently the most common form of digestive system cancer. According to updated cancer statistics, over 1.9 million new cases of CRC were diagnosed in 2020, resulting in 93,500 deaths.^1^ This accounts for approximately one‐tenth of all cancer cases and deaths. The incidence of CRC has increased from fifth to second place worldwide between 2018 and 2020.^1^, ^2^ Given the circumstances, enhancing the prevention, and screening of CRC is a crucial priority strategy. Previous studies have provided sufficient evidence that smoking,^3^ alcohol consumption,^4^ Type 2 diabetes (T2D),^5^ body mass index (BMI),^6^ waist‐to‐hip ratio (WHR),^6^ and total cholesterol (TC)^7^ are common risk factors for CRC. Nonetheless, limited research has been conducted on metabolic changes in CRC.

In recent years, the emergence of metabolomics as a component of systems biology has provided a novel approach to investigating the mechanisms underlying diseases. Specifically, metabolomics can provide insight into the biological mechanisms of diseases by identifying modified metabolites or metabolic pathways.^8^, ^9^ Over the past decades, there is a growing body of evidence that metabolic reprogramming and energy metabolism are crucial for the proliferation and metastasis of cancer cells.^10^, ^11^ For normal cells, while the altered metabolism provides support for cell proliferation or division, it can also affect cell differentiation making the cells predisposed to cancer.^10^ In addition, targeted regulation of metabolites has potential applications in the treatment of cancer. The most noteworthy example of cancer treatment through targeted regulation of metabolism is dichloroacetate (DCA). Previous studies provide convincing evidence that DCA can cut off pyruvate dehydrogenase (PDH) phosphorylation^12^ and reverse the Warburg effect to boost mitochondrial pyruvate oxidation to inhibit tumor proliferation.^13^, ^14^ Regulating cellular metabolism has been shown to increase the sensitivity of cancer cells to treatment.^15^ The combination of cellular metabolism inhibitors is considered a promising strategy to overcome the chemotherapy resistance, presenting a potential avenue for future research.

Therefore, exploring the metabolites associated with the development of CRC not only contributes to early screening and prevention of CRC, but also helps to understand the biological mechanisms of CRC for treatment. Unfortunately, the causality between metabolites and CRC is unclear, as there are no prospective studies of metabolites and CRC to date. The unavoidable design weaknesses of conventional observational studies, such as altered metabolites due to deliberate lifestyle changes in patients after the cancer diagnosis, chronic intake of certain drugs, and tumor cell‐induced changes in metabolic substances, resulted in an ambiguous causal relationship between metabolites and CRC. Rigorous randomized controlled trials (RCTs) have the highest credibility in evidence‐based medicine to prove causal effects, but they are difficult to implement due to ethical issues, observation time, costly money, and other constraints. Hence, metabolites that increase the risk of CRC cannot be identified based on the existing evidence.

Mendelian randomization (MR) studies have recently gained widespread usage in the investigation of disease etiology. In the absence of RCTs, MR is the most compelling strategy to explore the causality between the exposure of interest and outcome.^16^ MR assessed the causal effects of genetically proxied exposure of interest on outcomes by selecting exposure‐associated single nucleotide polymorphisms (SNPs) as instrumental variables (IVs).^17^ Specifically, this IVs alternative approach mimics RCTs since SNPs are randomly assigned to offspring at the time of conception, which largely avoids confounding factors because sex and age are less likely to bias the causal effect.^18^ Similarly, the reverse causality caused by MR studies is less likely since genotype formation is before the disease.

Since there is a lack of understanding related to the causality between blood metabolites and CRC, further research is required in this area. In this work, we performed MR analysis to comprehensively explore the causal effects of 486 blood metabolites on CRC via genome‐wide association study (GWAS) summary data. Furthermore, we conducted colocalization analysis and metabolic pathway analysis to investigate the underlying biological processes of CRC at the gene and protein levels. This study aims to reveal the metabolism‐related etiology of CRC and to provide insight into its biological processes.

## 2 METHODS AND MATERIALS

### 2.1 Study design

**FIGURE 1:**
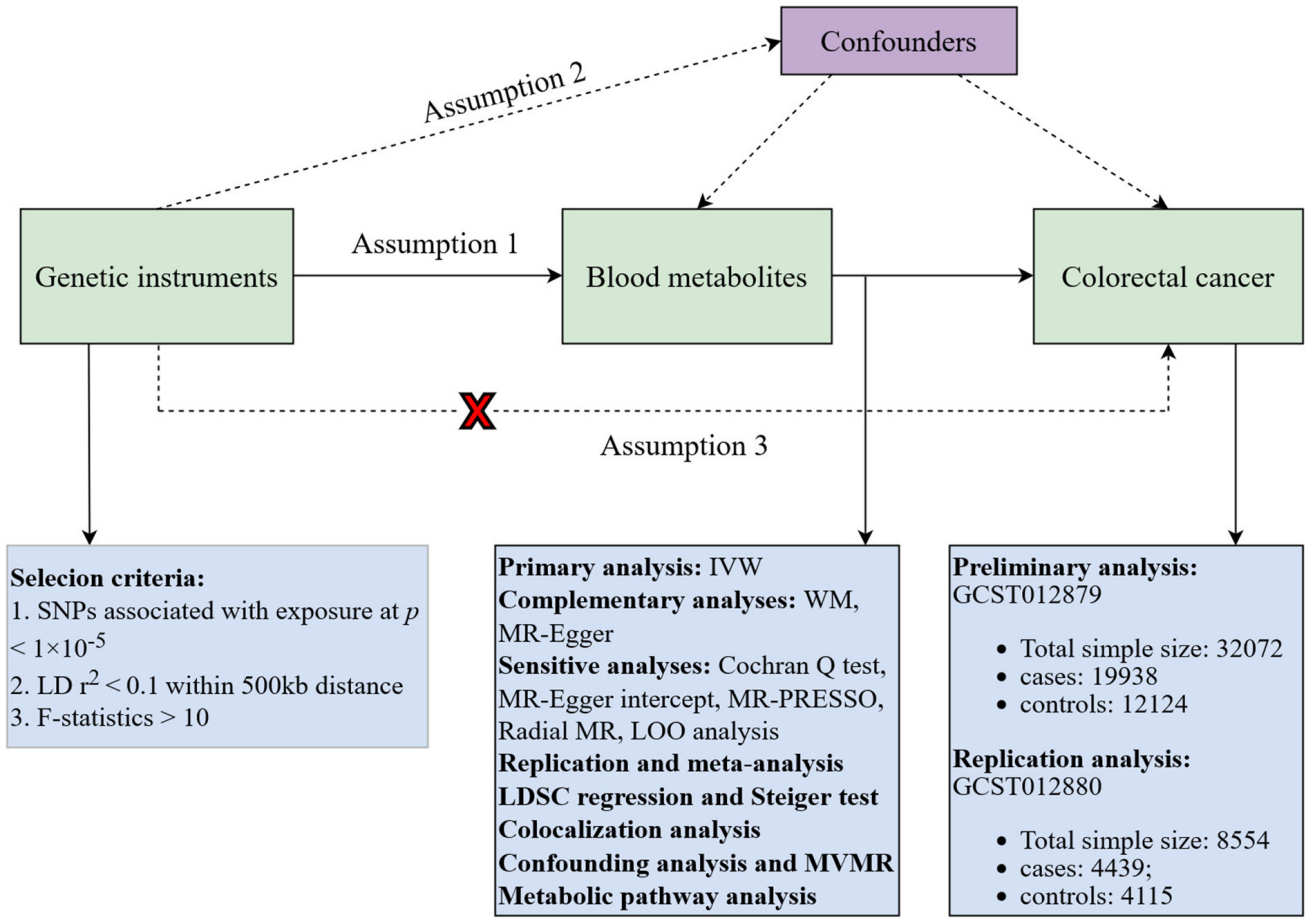
Overview of this Mendelian randomization (MR) analysis. Assumption 1, genetic instruments are strongly associated with the exposures of interest; Assumption 2, genetic instruments are independent of confounding factors; Assumption 3, genetic instruments are not associated with outcome and affect outcome only via exposures. IVW, inverse variance weighted; LD, linkage disequilibrium; LDSC, linkage disequilibrium score; LOO analysis, leave‐one‐out analysis; MR‐PRESSO, MR‐Pleiotropy RESidual sum and outlier; MVMR, multivariable Mendelian randomization analysis; SNPs, single nucleotide polymorphisms; WM, weighted median.

A valid MR study should comply with three assumptions: (1) IVs are strongly associated with the exposures of interest; (2) IVs are independent of confounding factors; (3) IVs are not associated with outcome and affect outcome only via exposures.^19^ All MR analyses in this study were performed in R software (4.2.1) by Two Sample MR, MRPRESSO, and Radial MR packages. An overview of the study is shown in Figure 1. This study was designed with reference to the MR study by Cai et al.^20^

### 2.2 GWAS data for 486 blood metabolites and CRC

Genetic data for blood metabolites were accessed from the metabolomics GWAS server (https://metabolomics.helmholtz‐muenchen.de/gwas/). Notably, this is the most comprehensive report to date on the genetic loci of blood metabolites, which eventually identified almost 2.1 million SNPs for 486 metabolites associated with human genetic variants by Genome‐wide association scans with high‐throughput metabolic profiling conducted by Shin et al.^21^ The detailed names of 486 metabolites are presented in Table S1, where the chemical properties of the metabolites named with X‐ are unknown. Specifically, the study included 7824 European, including 1768 from the KORA F4 study in Germany and 6056 from the UK Twin Study. Among the 486 metabolites, 107 were defined as unknown due to as yet poorly defined chemical properties. Another 309 metabolites were chemically authenticated and allocated to eight broad metabolic groups, including amino acid, carbohydrate, cofactors and vitamin, energy, lipid, nucleotide, peptide, and xenobiotic metabolism, as documented in the Kyoto Encyclopedia of Genes and Genomes (KEGG) database.^22^

GWAS summary data for CRC download from GWAS Catalog (https://www.ebi.ac.uk/gwas/)^23^ on November 5, 2022 and the GWAS Catalog accession number is GCST012879. Specifically, the GWAS data containing 39,216,056 SNPs were derived from a meta‐analysis of previous GWAS studies related to CRC conducted by Huyghe and colleagues, with a total sample size of 32,072 Europeans containing 19,938 cases and 12,124 controls.^24^ More detailed information about the GWAS data can be obtained from the study of Huyghe et al.^24^ The GWAS data for CRC mentioned above were used for preliminary analysis.

### 2.3 IVs selection

To satisfy assumption (1), IVs associated with blood metabolites were identified by rigorous screening conditions from multiple perspectives. Given the modest number of metabolite‐associated SNPs, we eased the significance threshold *p* < 1 × 10^‐5^ to select SNPs related to metabolites. Then, we clumped SNPs by removing linkage disequilibrium (LD, *R* ^2^ > 0.1 and within 500 kb). This criterion has been widely applied in previous studies.^25^, ^26^ To eliminate the bias induced by poor instruments, we calculated *R* ^2^ and F statistics for each SNP. *R* ^2^ and *F* statistics are calculated as follows:

$$\begin{array}{c} R^{2}=\frac{2*\beta^{2}*\mathrm{EAF}*\left(1-\mathrm{EAF}\right)}{\left[2*\beta^{2}*\mathrm{EAF}*\left(1-\mathrm{EAF}\right)+2*{\left(\mathrm{se}\;\left(\beta \right)\right)}^{2}*N*\mathrm{EAF}*\left(1-\mathrm{EAF}\right)\right]};\\ F=\frac{N-k-1}{k}*\frac{R^{2}}{1-R^{2}} \end{array}$$

where β is the effect size for the genetic variant of interest; EAF is the effect allele frequency for the genetic variant of interest; se (β) is the standard error of effect size for the genetic variant of interest; *R* ^2^ is the instrumental variable that explains the degree of exposure (determinant coefficient of regression equation); *N* is the sample size of the exposure; *k* is the number of SNPs (instrumental variants). SNPs with *F* < 10 were defined as poor genetic variants and were removed.^18^ Next, we extracted metabolite‐associated SNPs from the outcome and discarded SNPs associated with the outcome (*p* < 1 × 10^−5^). We further harmonized SNPs for exposure and outcome, and palindromic effects and allelic inconsistent SNPs were removed (e.g. A/G vs. A/C). Then, to satisfy assumption (3), we removed outcome‐related SNPs (*p* < 1 × 10^–5^) in the IVs. Finally, we performed MR analysis on metabolites with more than two SNPs.^27^

### 2.4 Statistical analysis and sensitivity analysis

The causal effect of blood metabolites and CRC was primarily assessed based on the results of random‐effect inverse variance weighted (IVW). Because IVW estimates are derived from a summary analysis of Wald ratios for all genetic variants.^28^ IVW is based on the assumption that there is no horizontal pleiotropy for all SNPs, under this premise IVW provides the most accurate assessment of causal effects.^28^Hence, we initially screened for blood metabolites with causal effects on CRC using IVW‐based estimates. To acquire more reliable results, we applied other two methods to further evaluate the metabolites with significant estimates (IVW derived *p* < 0.05). MR‐Egger and weighted median (WM) methods were defined as complementary analyses. These two methods can provide more robust estimates under lenient conditions. WM allows less than 50% of SNPs to be invalid, while MR‐Egger provides horizontal pleiotropy and heterogeneity detection in the presence of horizontal pleiotropy for all SNPs.^28^, ^29^ MR‐Egger regression can provide unbiased estimates when consistent with the InSIDE assumption (Strength of IVs independent of direct effects).^29^

Sensitivity analysis is essential because it examines the horizontal pleiotropy and heterogeneity that can severely violate MR estimates. Horizontal pleiotropy is observed when IVs influence the outcome through other pathways than the exposure of interest. As such, we performed several tests to ensure convincing estimates. In this study, we utilized four methods to detect and correct for heterogeneity and pleiotropy, including the Cochran Q test, MR‐Egger intercept test, MR‐Pleiotropy RESidual Sum and Outlier (MR‐PRESSO), and Radial MR. Cochran Q test‐derived *p* < 0.05 was considered as heterogeneity of the results.^30^ MR‐Egger intercept was calculated to test for directional pleiotropy and bias due to invalid IVs.^29^ Subsequently, Radial MR^31^ was performed to identify outliers, and MR analysis was repeated after eliminating heterogeneous SNPs. Ultimately, we use MR‐PRESSO^32^ to check again for the presence of heterogeneous SNPs. In terms of the robustness of the results, we performed a leave‐one‐out (LOO) analysis which assesses whether the results are heavily influenced by a single SNP by discarding each SNP in turn and then performing MR analysis.^29^

In a word, we rigorously screened blood metabolites with potential causal effects on CRC by multiple criteria: (1) *p* value for the primary analysis was significant (IVW derived *p* < 0.05). (2) Consistent direction and magnitude within the three MR methods. (3) No heterogeneity or horizontal pleiotropy in MR results. (4) MR estimates are not severely disturbed by a single SNP.

Moreover, to assess the statistical power of the estimates, we calculated the power with an online website (https://shiny.cnsgenomics.com/mRnd/).^33^ Specifically, this tool calculates power values based on asymptotic theory to detect causal effects derived from IVs. We set the Type I error rate is 0.05 and calculated power using *R* ^2^ of IVs, the proportion of cases with an outcome, and the odds ratio (OR) derived from IVW analysis.

### 2.5 Replication and meta‐analysis

To comprehensively estimate the robustness of the candidate metabolites identified based on the above criteria, we replicated the IVW analysis in an additional CRC cohort that included 4439 European ancestry cases and 4115 European ancestry controls.^24^ The GWAS data for CRC in the replication analysis was also obtained from GWAS Catalog and the accession number is GCST012880. Specifically, this GWAS data is part of the results of a meta‐analysis of 30 existing GWAS studies for CRC. The study included 8554 Europeans from the Germany, Netherlands, United States, and Nauru and identified 39,216,056 SNPs for CRC. In brief, GWAS data with the accession number GCST012879 is used for preliminary analysis while GWAS data with accession number GCST012880 is used for replication analysis. We finally determined the blood metabolites with causal effects on CRC by the results of a meta‐analysis of two MR analyses. Meta‐analysis was implemented based on a random effects IVW model on Review Manager 5.4 software.

### 2.6 Evaluation of genetic correlation and directionality

However, MR estimates can violate causal effects under the premise of genetic correlation between exposure and outcome of interest.^34^, ^35^ Although SNPs related to CRC were excluded in the selection of IVs, SNPs with no relation may also mediate the genetics of CRC. Linkage disequilibrium score (LDSC) regression can estimate coinheritance by performing Chi‐squared statistics for two traits based on SNP. Hence, to ensure causal effects were not confused by the coheritability of exposure with the outcome, LDSC was implemented to check the genetic correlation between the screened metabolites and CRC.

In addition, we further performed the Steiger test to reject the resulting bias caused by reverse causality.^36^ The direction of causal inference might be false in the presence of explained variance of IVs in CRC is stronger than blood metabolites.

### 2.7 Colocalization analysis

To further investigate whether the associations of the identified metabolites between CRC were driven by a locus within a genomic region, we performed colocalization analysis using the coloc R package.^37^ Genes with important regulatory effects on metabolites were identified from previous studies. The cis‐expression quantitative trait loci (eQTL) and protein quantitative trait loci (pQTL) summary‐level data for genes expression in blood were obtained from the eQTLGen Consortium including 31,684 blood samples (https://www.eqtlgen.org/)^38^ and a large‐scale pQTL study in 35,559 Icelanders,^39^ respectively. This colocalization analysis can pinpoint the causal association between metabolites and CRC to the same causal variant locus within a specific genomic region. For each locus of variation, the coloc method assessed the posterior probability (H0, H1, H2, H3, and H4) of the following five hypotheses in the Bayesian framework: (1) no association with either trait; (2) association with Trait 1 only; (3) association with Trait 2 only; (4) both traits are associated, but distinct causal variants were for two traits; and (5) both traits are associated, and the same shares causal variant for both traits.^40^ The colocalization analysis was performed using the default priors (*p*1 = 1 × 10^−4^, *p*2 = 1 × 10^−4^, and *p*12 = 1 × 10^−5^). PP.H4 > 80% colocalization analysis (H4) results provide strong evidence supporting the existence of shared causal variants within specific genomic regions affecting gene expression and CRC risk.

### 2.8 Confounding analysis and multivariable MR analysis

Although we assessed the horizontal pleiotropy of the MR results through a series of sensitivity analyses to detect any SNPs that violated the MR assumptions,^2^ there may also be little residual confounding SNPs. We checked IVs for metabolites at the Phenoscanner V2 website (http://www.phenoscanner.medschl.cam.ac.uk/) to evaluate whether each SNP was associated with known risk factors for CRC, such as smoking,^3^ alcohol consumption,^4^ T2D^5^ BMI,^6^ WHR,^6^ and TC.^7^ If any SNPs were observed to be associated with the above confounding factors (*p* < 1 × 10^−5^), MR analysis would be re‐performed after removing these SNPs to verify the reliability of the results.

To avoid IVs violating Assumptions 2 and 3 of MR, MR analysis is required to ensure that genetic variants are associated with a single risk factor. In practice, however, some genetic variants are associated with multiple risk factors, which is described as pleiotropy.^41^ In this case, multivariable MR (MVMR) can correct for interactions of genetic variation between exposures by incorporating multiple exposures that may interact with each other. In brief, univariable MR assessed the total effect of exposure on outcome, and MVMR assessed the direct effect of each exposure (independent of any other exposures) on outcome.^41^ In this study, we performed MVMR on the identified metabolites to adjust for their interactions. MVMR was performed using IVW^42^ and MR‐PRESSO.^32^ The IVW method of multivariate MR is to regress all exposed SNPs with the outcome, weighting for the inverse variance of the outcome. MRPRESSO can remove outliers to correct for the pleiotropy of IVs.

### 2.9 Metabolic pathway analysis

To specify the biological mechanisms involved in blood metabolites that have causal effects on CRC, we further performed metabolic pathway analyses using the MetaboAnalyst 5.0 (https://www.metaboanalyst.ca/)^43^ to explore the potential pathogenesis of CRC.

## 3 RESULTS

### 3.1 Preliminary analysis

**FIGURE 2:**
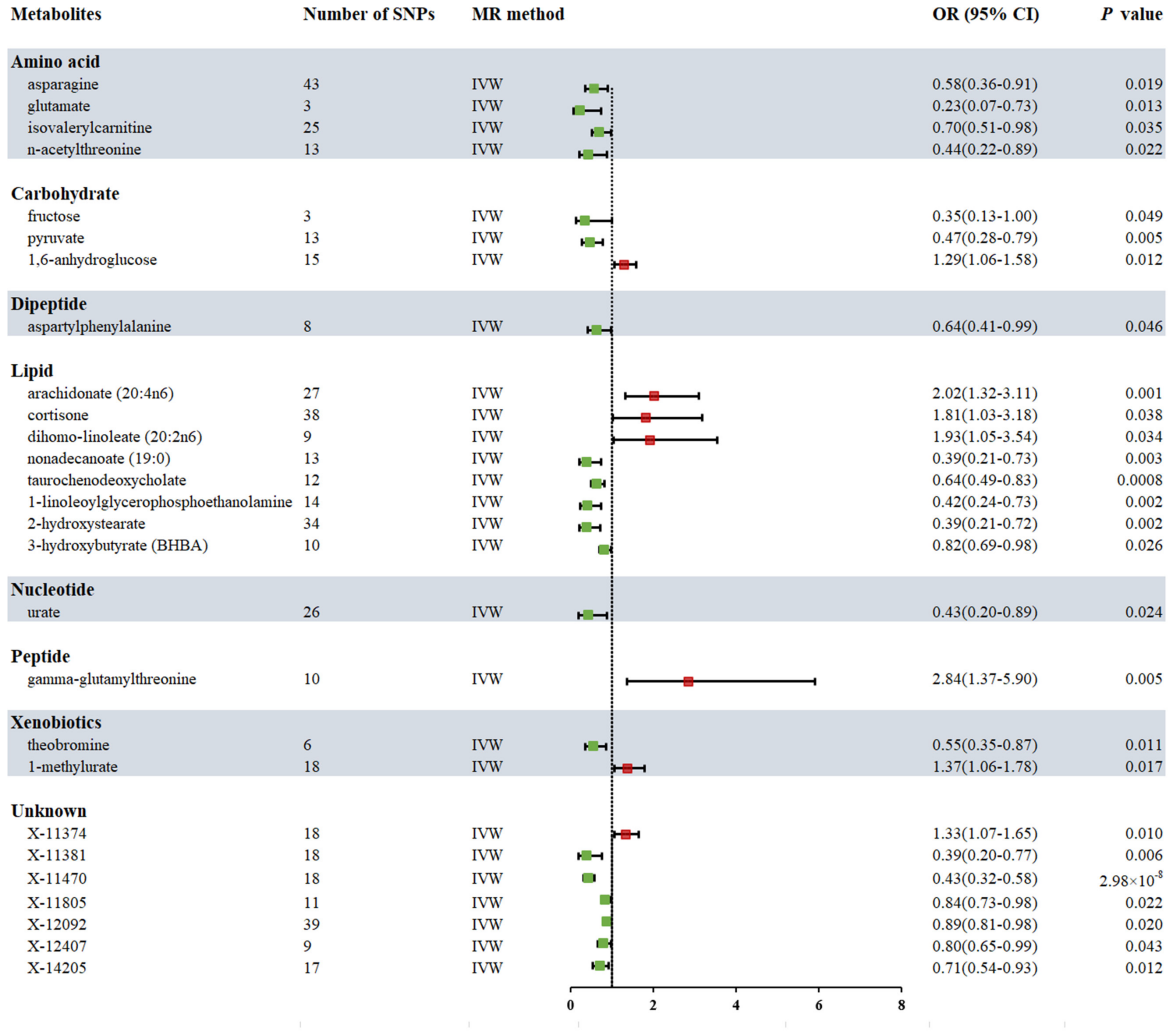
Forest plot for the causality of blood metabolites on colorectal cancer derived from inverse variance weighted (IVW) analysis. CI, confidence interval; IVW, inverse variance weighted; OR, odds ratio; SNPs, single nucleotide polymorphisms.

**FIGURE 3:**
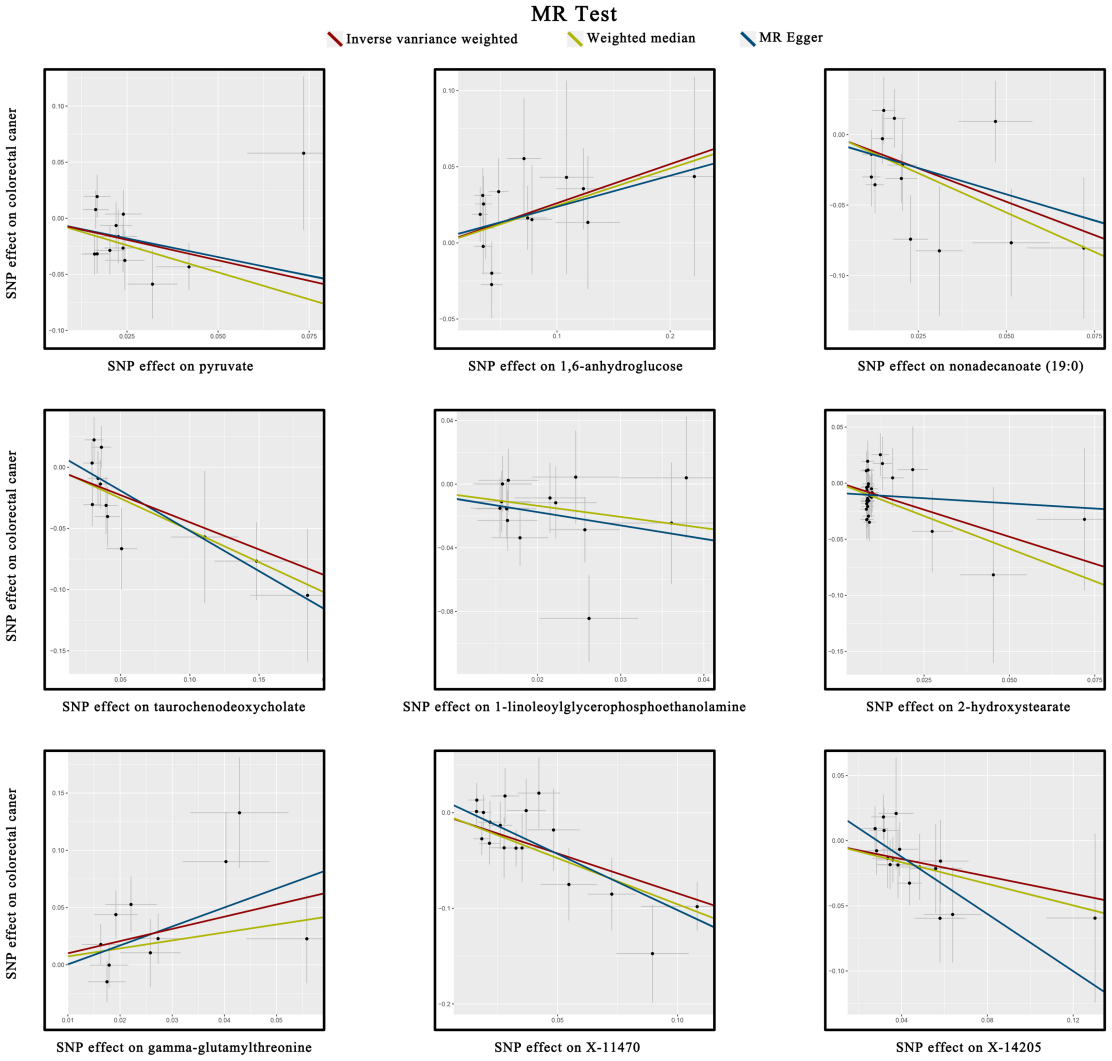
Scatterplot of significantly associated (IVW derived *p* < 0.05) and directionally consistent estimates. SNP, single nucleotide polymorphisms.

After tightly controlling the quality of IVs, 486 metabolites were eventually captured in the MR study. The filtered IVs contained SNPs ranging from 3 to 501 (genetically proxied for glutamate consisted of 3 SNPs; 2‐methoxyacetaminophen sulfate generated the most genetically proxied: 501 SNPs). The F statistics of all SNPs associated with metabolites were greater than 10, which indicated a strong power of IVs. The detailed data of IVs are presented in Table S2. All outliers are identified and removed by Radial MR prior to MR analysis (Table S3). Then IVW analysis was performed to preliminarily identify 27 metabolites with potential causal effects on CRC, including 20 metabolites whose chemical identity is known and seven metabolites whose chemical identity is unknown (Figure 2). As shown in Figure 2, the 20 known metabolites were chemically assigned to the amino acid, carbohydrate, dipeptide, lipid, nucleotide, peptide, and xenobiotics. After combining complementary and sensitivity analyses, nine eligible metabolites that met the rigorous screening criteria were identified as candidates (Table 1), including pyruvate (OR 0.47, 95% confidence interval (CI): 0.28–0.79, *p* = 0.005), 1,6‐anhydroglucose (OR 1.29, 95% CI: 1.06–1.58, *p* = 0.012), nonadecanoate (19:0) (OR 0.39, 95% CI: 0.21–0.73, *p* = 0.003), taurochenodeoxycholate (OR 0.64, 95% CI: 0.49–0.83, *p* = 0.0008), 1‐linoleoylglycerophosphoethanolamine (OR 0.42, 95% CI: 0.2–0.73, *p* = 0.002), 2‐hydroxystearate (OR 0.39, 95% CI: 0.21–0.72, *p* = 0.002), gamma‐glutamylthreonine (OR 2.84, 95% CI: 1.37–5.90, *p* = 0.005), X‐11470 (OR 0.43, 95% CI: 0.32–0.58, *p* = 2.98 × 10^−08^), X‐14205 (OR 0.71, 95% CI: 0.54–0.93, *p* = 0.012). In summary, the estimates derived from IVW are significant (*p* < 0.05) while the direction and magnitude of IVW, MR‐Egger, and WM estimates were consistent (Figure 3). After removing the outliers, the MR‐PRESSO results also did not favor the existence of heterogeneous SNPs (Table S4). The Cochran *Q* test (*p* > 0.05) and MR‐Egger intercept test (*p* > 0.05) provided strong evidence for the absence of heterogeneity and pleiotropy (Table 1). LOO analysis results supported that a single SNP did not cause bias in MR estimation (Figure S1). The statistical power of all estimates is >0.8 (Table 1). These nine blood metabolites are considered to be candidates for further analysis.

**TABLE 1:** Supplementary and sensitivity analyses for causality from blood metabolites on colorectal cancer.

Metabolites	*N*	MR analysis			Heterogeneity		Pleiotropy		
		Methods	OR (95% CI)	*p*	Q	*p*	Intercept	*p*	Power
Amino acid									
Asparagine	43	ME	0.40(0.17–0.96)	0.047	35.19	0.76	0.005	0.35	1.00
		WM	0.50(0.25–1.00)	0.053					
Glutamate	3	ME	0.0003(4 × 10^−11^‐2600.78)	0.503	0.67	0.72	0.116	0.57	1.00
		WM	0.18(0.04–0.79)	0.023					
Isovalerylcarnitine	25	ME	0.47(0.25–0.89)	0.030	16.19	0.88	0.011	0.17	1.00
		WM	0.60(0.37–0.98)	0.041					
*N*‐acetylthreonine	13	ME	0.17(0.02–1.38)	0.127	11.24	0.51	0.017	0.37	1.00
		WM	0.39(0.14–1.10)	0.075					
Carbohydrate									
Fructose	3	ME	0.05(0.001–1.77)	0.347	1.33	0.51	0.052	0.46	1.00
		WM	0.26(0.07–0.94)	0.041					
Pyruvate	13	ME	0.53(0.12–2.42)	0.429	13.17	0.36	−0.003	0.88	1.00
		WM	0.38(0.18–0.79)	0.010					
1,6‐anhydroglucose	15	ME	1.22(0.82–1.82)	0.335	10.41	0.73	0.004	0.76	0.99
		WM	1.28(0.95–1.71)	0.105					
Dipeptide									
Aspartylphenylalanine	8	ME	0.87(0.27–2.77)	0.822	8.07	0.33	−0.012	0.59	1.00
		WM	0.63(0.36–1.12)	0.118					
Lipid									
Arachidonate (20:4n6)	27	ME	2.58(1.18–5.60)	0.025	20.09	0.79	−0.006	0.47	1.00
		WM	3.07(1.65–5.74)	0.0004					
Cortisone	38	ME	3.18(0.91–11.06)	0.077	24.87	0.94	−0.007	0.33	1.00
		WM	2.42(1.05–5.53)	0.037					
Dihomo‐linoleate (20:2n6)	9	ME	0.85(0.12–5.94)	0.878	7.47	0.49	0.019	0.42	1.00
		WM	1.57(0.67–3.68)	0.304					
Nonadecanoate (19:0)	13	ME	0.48(0.14–1.68)	0.276	15.09	0.24	−0.006	0.70	1.00
		WM	0.33(0.14–0.75)	0.008					
Taurochenodeoxycholate	12	ME	0.52(0.33–0.80)	0.015	12.41	0.33	0.014	0.27	1.00
		WM	0.60(0.42–0.85)	0.004					
1‐linoleoylglycerophosphoethanolamine	14	ME	0.42(0.05–3.56)	0.442	9.95	0.70	−9.47E‐06	1.00	1.00
		WM	0.50(0.24–1.07)	0.076					
2‐hydroxystearate	34	ME	0.84(0.19–3.76)	0.817	22.71	0.91	−0.009	0.28	1.00
		WM	0.31(0.13–0.75)	0.009					
3‐hydroxybutyrate (BHBA)	10	ME	0.90(0.71–1.16)	0.451	3.56	0.94	−0.012	0.33	0.84
		WM	0.82(0.66–1.02)	0.081					
Nucleotide									
Urate	26	ME	0.26(0.05–1.28)	0.111	29.37	0.25	0.007	0.50	1.00
		WM	0.25(0.09–0.68)	0.007					
Peptide									
Gamma‐glutamylthreonine	10	ME	5.18(0.67–39.87)	0.153	12.93	0.17	−0.016	0.55	1.00
		WM	2.02(0.83–4.89)	0.120					
Xenobiotics									
Theobromine	6	ME	0.30(0.009–9.59)	0.534	4.50	0.48	0.021	0.75	1.00
		WM	0.62(0.34–1.14)	0.125					
1‐methylurate	18	ME	1.48(0.95–2.32)	0.103	9.06	0.94	−0.004	0.68	1.00
		WM	1.45(1.02–2.07)	0.040					
Unknown									
X‐11374	18	ME	1.67(1.04–2.69)	0.050	9.61	0.92	−0.013	0.30	1.00
		WM	1.44(1.05–1.96)	0.023					
X‐11381	18	ME	1.02(0.15–6.97)	0.987	11.23	0.84	−0.016	0.32	1.00
		WM	0.46(0.19–1.22)	0.087					
X‐11470	18	ME	0.31(0.19–0.52)	0.0003	14.08	0.66	0.015	0.14	1.00
		WM	0.38(0.23–0.63)	0.0002					
X‐11805	11	ME	0.83(0.66–1.04)	0.148	10.57	0.39	0.002	0.88	1.00
		WM	0.82(0.67–1.01)	0.060					
X‐12092	39	ME	0.89(0.78–1.01)	0.089	22.12	0.98	0.0007	0.89	1.00
		WM	0.91(0.81–1.03)	0.129					
X‐12407	9	ME	0.79(0.45–1.37)	0.434	3.30	0.91	0.001	0.95	0.89
		WM	0.82(0.63–1.07)	0.152					
X‐14205	17	ME	0.33(0.14–0.81)	0.029	9.49	0.89	0.032	0.10	1.00
		WM	0.66(0.46–0.95)	0.025					

Abbreviations: CI, confidence interval; ME, MR‐Egger; N, number of single nucleotide polymorphisms; OR, odds ratio; WM, weighted median.

### 3.2 Replication and meta‐analysis

**FIGURE 4:**
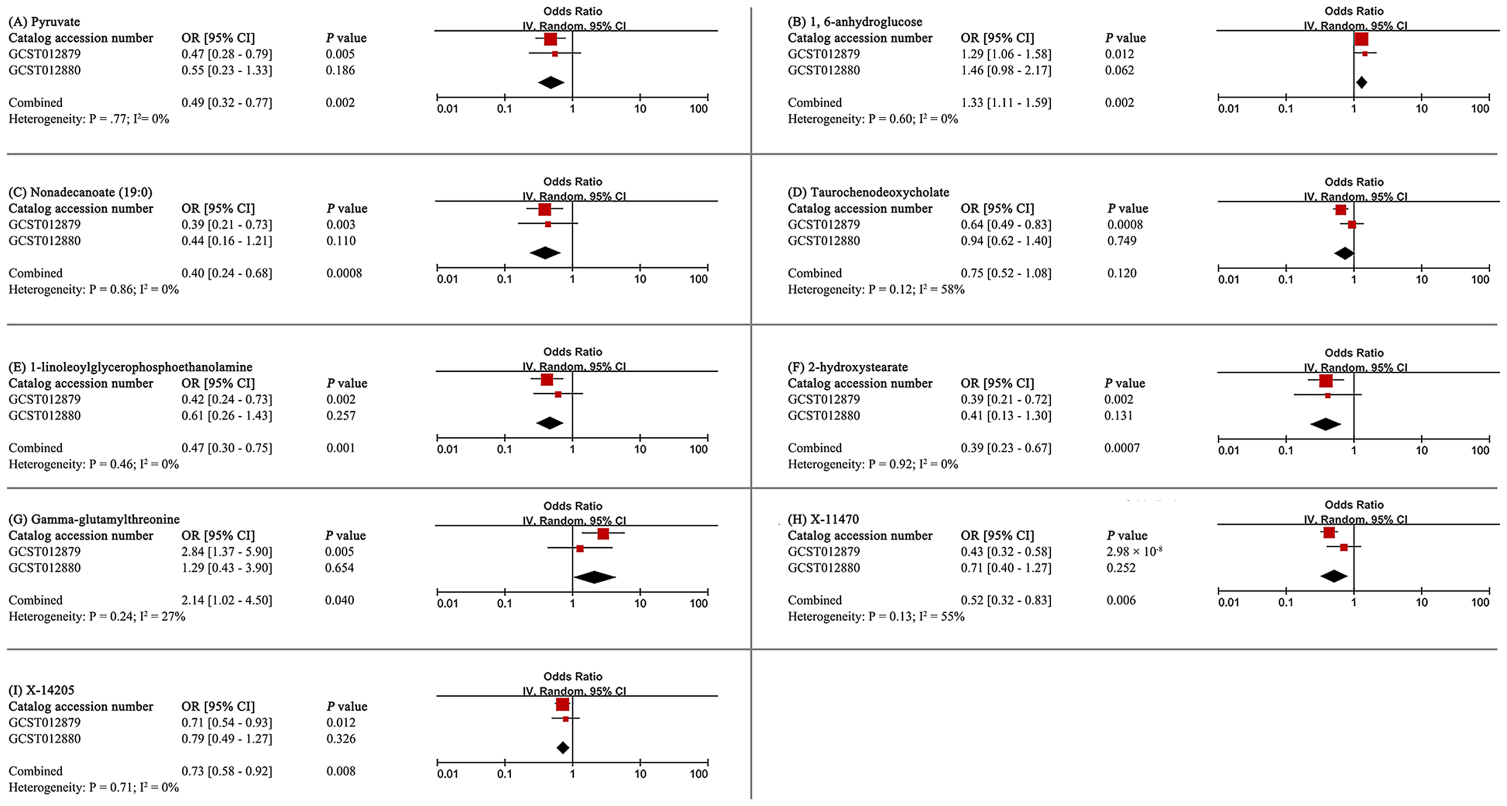
Meta‐analysis of significantly associated (IVW derived *p* < 0.05) between metabolites and colorectal cancer. 95% CI, 95% confidence interval; OR, odds ratio.

To enhance the persuasiveness of the estimates, we replicated the MR analysis with another GWAS data for CRC. As would be expected, similar trends were observed for candidate metabolites in another GWAS data for CRC, although the results were not significant due to the enormous difference in sample size. The results of the meta‐analysis further determined that eight blood metabolites (six known and two unknown) can affect CRC (Figure 4). In detail, genetic susceptibility for higher levels of pyruvate (OR 0.49, 95% CI: 0.32–0.77, *p* = 0.002), nonadecanoate (19:0) (OR 0.40, 95% CI: 0.24–0.68, *p* = 0.0008), 1‐linoleoylglycerophosphoethanolamine (OR 0.47, 95% CI: 0.30–0.75, *p* = 0.001), 2‐hydroxystearate (OR 0.39, 95% CI: 0.23–0.67, *p* = 0.0007), X‐11470 (OR 0.52, 95% CI: 0.32–0.83, *p* = 0.006), X‐14205 (OR 0.73, 95% CI: 0.58–0.92, *p* = 0.008) decreased risk of CRC, while genetic liability for higher levels of 1,6‐anhydroglucose (OR 1.33, 95% CI: 1.11–1.59, *p* = 0.002), gamma‐glutamylthreonine (OR 2.14, 95% CI: 1.02–4.50, *p* = 0.040) promoted susceptibility to CRC. Even though it showed a consistent direction in both MR analyses, taurochenodeoxycholate was discarded due to the meta‐analysis revealed non‐significant estimates (OR 0.75, 95% CI: 0.52–1.08, = 0.120).

### 3.3 Evaluation of genetic correlation and directionality

LDSC‐based estimates noted little genetic correlation observed between CRC and pyruvate (R_g_ = 0.259, Se = 0.218, *p* = 0.236), 1,6‐anhydroglucose (R_g_ = 0.285, Se = 0.304, *p* = 0.348) nonadecanoate (19:0) (R_g_ = 0.169, Se = 0.186, *p* = 0.363), 1‐linoleoylglycerophosphoethanolamine (R_g_ = −0.003, Se = 1.636, *p* = 0.999), 2‐hydroxystearate (R_g_ = 0.098, Se = 0.134, *p* = 0.466), gamma‐glutamylthreonine (R_g_ = −0.017, Se = 0.268, *p* = 0.950), X‐11470 (R_g_ = 0.273, Se = 0.286, *p* = 0.340), X‐14205 (R_g_ = 0.109, Se = 0.380, *p* = 0.774) suggesting that MR estimates are not confounded by shared genetic components. Subsequently, we estimated the SNP‐heritability of eight metabolites based on LDSC, and the SNP‐heritability (proportion of variance attributed to genome‐wide SNPs) of metabolites ranged from 0.0318 (1‐linoleoylglycerophosphoethanolamine) to 0.1601 (gamma‐glutamylthreonine) (Table S5). In addition, the Steiger test revealed that the causality between genetically proxied metabolites and CRC was not violated by reverse causal effects (Table S6).

### 3.4 Colocalization analysis

For the six known metabolites that have been identified in this study that are associated with CRC risk, we searched for relevant targets of metabolites in previously published studies. We found that lactate dehydrogenase A (LDHA) regulated pyruvate metabolism and was significantly associated with the development of cancer,^44^, ^45^ while the eQTL and pQTL of LDHA in blood were also available in publicly available databases. Few targets with important regulatory effects on other metabolites have been reported. Therefore, we only performed colocalization analysis of LDHA from the gene and protein level. We accessed whole blood LDHA eQTL and pQTL signals at genome‐wide significant levels (*p* < 5 × 10^−8^) from the eQTL consortium and a large‐scale GWAS on blood proteome (Table S7,**S8**). Colocalization analysis results from eQTL (PP.H4 = 99.59%, SNP = rs6486426) and pQTL (PP.H4 = 99.85%, SNP = rs116841148) strongly support that LDHA expression and CRC risk are driven by the shared causal variant loci. Specifically, significant loci on GWAS signaling can increase CRC risk by influencing the biological process of LDHA expression.

### 3.5 Confounding analysis and MVMR

Although SNPs that violated the estimates have been excluded by sensitivity analysis in this work, to satisfy assumption^2^ (IVs are independent of confounding factors), we checked whether all SNPs associated with eight metabolites were independent of common risk factors (smoking, alcohol consumption, T2D, BMI, and WHR) for CRC in the Phenoscanner one by one. We found gamma‐glutamylthreonine to be independent of any confounding factors. Among the other IVs of seven metabolites, a total of 13 SNPs were observed to be associated with common risk factors for CRC (Table S9). As expected, after removing these SNPs, the estimates remained significant: pyruvate (OR 0.53, 95% CI: 0.30–0.93, *p* = 0.027), 1,6‐anhydroglucose (OR 1.31, 95% CI: 1.06–1.61, *p* = 0.011), nonadecanoate (19:0) (OR 0.35, 95% CI: 0.19–0.66, *p* = 0.001), 1‐linoleoylglycerophosphoethanolamine (OR 0.49, 95% CI: 0.26–0.91, *p* = 0.025), 2‐hydroxystearate (OR 0.39, 95% CI: 0.21–0.72, *p* = 0.003), X‐11470 (OR 0.44, 95% CI: 0.32–0.60, *p* = 3.00 × 10^−7^), X‐14205 (OR 0.75, 95% CI: 0.56–1.00, *p* = 0.047).

**FIGURE 5:**
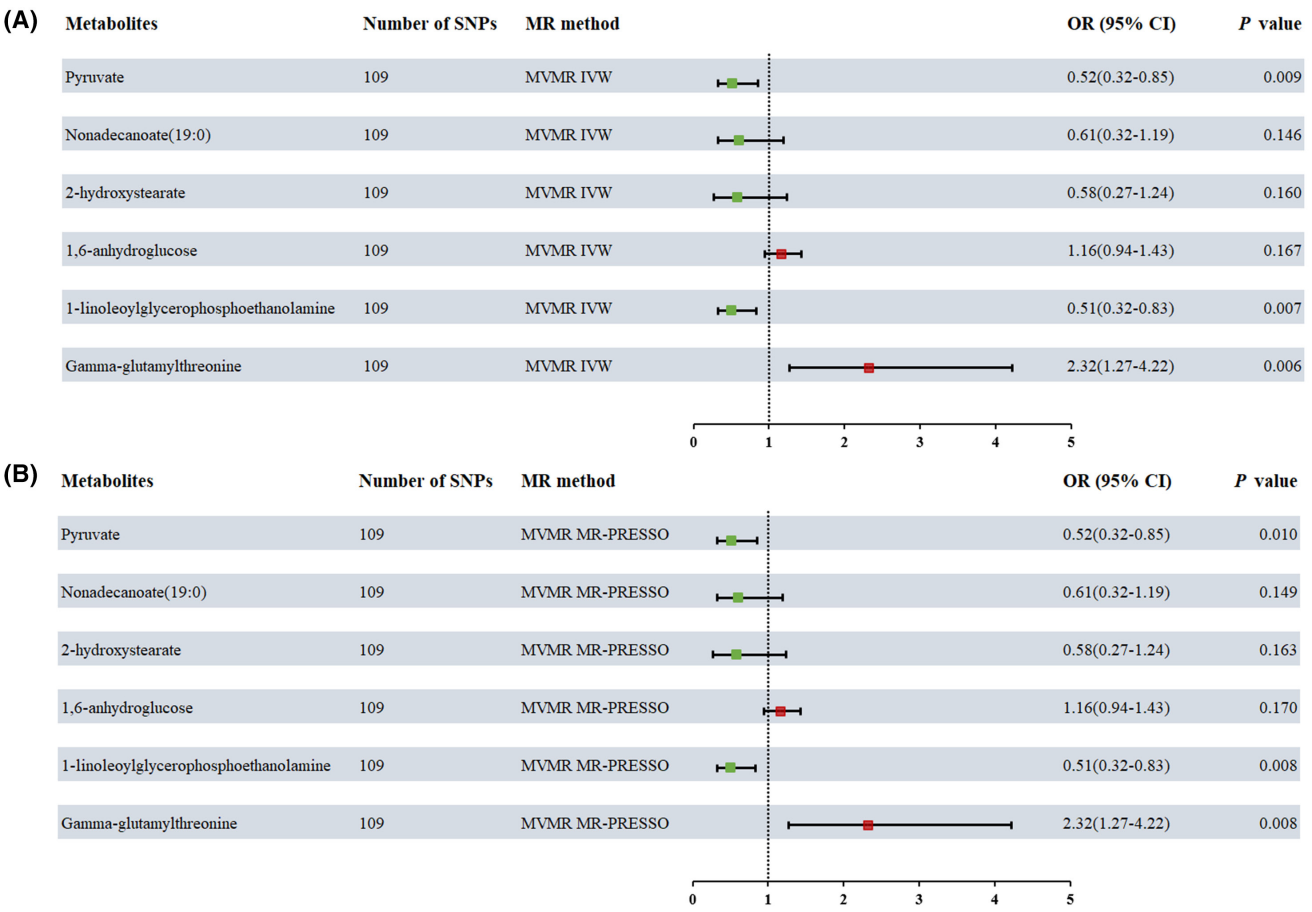
Multivariable MR analysis of the final identified blood metabolites. 95% CI, 95% confidence interval; IVW, inverse variance weighted; MVMR, Multivariable Mendelian randomization; MR‐PRESSO, MR‐Pleiotropy RESidual Sum and Outlier; OR, odds ratio.

After adjusting for metabolite interactions, MVMR estimates based on IVW (Figure 5A) and MR‐PRESSO (Figure 5B) methods both showed that genetically predicted pyruvate, 1‐linoleoylglycerophosphoethanolamine and gamma‐glutamylthreonine can directly influence CRC independently of other metabolites.

### 3.6 Metabolic pathway analysis

Based on six known metabolites, we identified nine metabolic pathways that may be involved in the mechanism of CRC pathogenesis (Table S10). Citrate cycle (TCA cycle), pyruvate metabolism, glycolysis/gluconeogenesis, alanine, aspartate and glutamate metabolism, glyoxylate and dicarboxylate metabolism, glycine, serine, and threonine metabolism, cysteine and methionine metabolism, arginine and proline metabolism, and tyrosine metabolism may be the potential biological mechanism for the development of CRC. Notably, pyruvate is involved in all metabolic pathways. This means that pyruvate and related metabolic pathways take an important role in the pathogenesis of CRC.

## 4 DISCUSSION

In the current work, we integrated two large‐scale GWAS data to explore the causal effects of genetically proxied 486 blood metabolites on CRC via a rigorous MR design. We finally determined that genetically determined high levels of pyruvate, nonadecanoate (19:0), 1‐linoleoylglycerophosphoethanolamine, 2‐hydroxystearate, X‐11470, and X‐14205 associated with lower CRC risk while genetically predisposition to high levels of 1,6‐anhydroglucose and gamma‐glutamylthreonine increased risk of CRC. MVMR estimates suggested that pyruvate, 1‐linoleoylglycerophosphoethanolamine and gamma‐glutamylthreonine can directly affect CRC independently of other metabolites. Colocalization analysis provided strong evidence that significant loci on GWAS signaling can increase CRC risk by influencing the expression of LDHA. Subsequently, we identified 9 metabolic pathways that may be involved in the biological mechanisms of CRC. To our knowledge, this is the first MR study to date that applied the most comprehensive blood metabolite GWAS data to explore the causality with CRC and incorporates metabolic pathways and colocalization analysis.

In recent years, the high incidence and mortality rates of CRC have put a heavy burden on people around the world, making early screening and prevention of CRC an urgent strategy. The emergence of metabolomics technologies has led to an increasing interest in exploring the perceived value of metabolites in CRC. Notably, blood metabolites visually provide a snapshot of biological mechanisms since they simultaneously capture endogenous and exogenous processes.^46^ For example, recent evidence suggests that tryptophan is metabolized by tumor cell‐secreted indoleamine‐2,3‐dioxygenase (IDO) and tryptophan‐2,3‐dioxygenase (TDO) catabolism to generate metabolites, particularly kynurenine.^47^ Depletion of tryptophan and accumulation of kynurenine induced T cell dysfunction and apoptosis resulting in immunosuppression.^47^, ^48^ Emerging IDO inhibitors are under clinical trials in a variety of cancers and are promising immunotherapeutic approaches applied to CRC.^48^, ^49^ Although previous studies have provided convincing evidence that metabolites are involved in the biological mechanisms of CRC, which are beneficial for CRC treatment, the contribution to early screening and prevention of CRC is limited due to the ambiguous causal relationship between the both. Therefore, we implemented a critical MR study in the hope that the causal relationship between blood metabolites and CRC and the metabolic pathways involved in them can be clarified, thus providing a reference direction for the screening and treatment of CRC.

In this work, we found that genetic sensitivity to high levels of pyruvate can keep the body away from CRC, while pyruvate is involved in nine significantly enriched metabolic pathways including the citrate cycle and pyruvate metabolism. Several previous studies are consistent with our results that pyruvate shows great opportunities for the prevention and treatment of cancer. Pyruvate bridges cytoplasmic and mitochondrial metabolism as a hub of cellular metabolism, and it mainly originates from the final process of glycolysis that pyruvate kinase encoding the conversion of phosphoenolpyruvate to pyruvate.^50^ Pyruvate in the cytosol is converted to lactate by lactate dehydrogenase (LDH), and a portion of pyruvate enters the mitochondrial matrix via the mitochondrial pyruvate carrier (MPC) and is converted by the PDH complex to produce acetyl coenzyme A and other substances to carry out the citrate cycle or to regulate cholesterol and lipid metabolism.^51^ Returning to the evidence from previous studies, we speculate that high levels of pyruvate may reduce the risk of CRC through the following pathways. The first and most important pathway is to reverse the Warburg Effect. The Warburg Effect was first reported by Warburg O et al. in 1927 and widely accepted that tumor cells rely more on glycolysis than mitochondrial phosphorylation for energy supply which is different from normal cells.^52^ One of the culprits of the Warburg Effect is the weakening of MPC activity.^53^ CRC is more glycolysis‐dependent in the early stages, while MPC activity is downregulated.^54^ High levels of pyruvate can increase MPC activity and thus enhance mitochondrial oxidative phosphorylation to reduce glycolysis and lactate production, which reverses the Warburg effect to some extent.^55^ Second, pyruvate is an endogenous anti‐inflammatory and anti‐oxidant molecule. Ramos‐Ibeas P et al. reported that pyruvate presented the best antioxidant stress effect on fibroblasts and embryonic stem cells, superior to traditional well‐known antioxidants such as selenium, N‐acetylcysteine, and Trolox.^56^ Pyruvate also inhibited the expression of various inflammatory factors such as tumor necrosis factor, NF‐kB pathway, and interleukin‐6 and promoted insulin secretion.^57^ As described, the anti‐oxidative and anti‐inflammatory effects of pyruvate support a microenvironment that is not conducive to cancer development. Ultimately, pyruvate can also reduce fat and alleviate insulin resistance. Ultimately, pyruvate can also reduce fat and alleviate insulin resistance. High levels of pyruvate promote the tricarboxylic acid cycle and reduce lactate synthesis. Lactate is a key mediator in PDH kinase‐induced adipogenesis and is associated with obesity and insulin resistance.^58^, ^59^ Therefore, adequate amounts of pyruvate may indirectly decrease the synthesis of fat and ameliorate obesity to lower the risk of CRC. To summarize, pyruvate appears to be a metabolite of great opportunity in the prevention and treatment of CRC.

This MR study also identified three other blood metabolites (nonadecanoate (19:0), 1‐linoleoylglycerophosphoethanolamine, and 2‐hydroxystearate) that have protective effects on CRC. Unfortunately, there are few previous reports of nonadecanoate‐related effects, and the relationship between nonadecanoate and cancer deserves further study. For 1‐linoleoylglycerophosphoethanolamine, Mika A et al. noted that phosphoethanolamine was highly expressed in CRC tissues but its contribution to CRC was not clear.^60^ Another prospective cohort study suggested that serum higher levels of linoleoylglycerophosphocholine decreased the risk of atherosclerosis and kidney failure.^61^ In addition, 1‐linoleoylglycerophosphoethanolamine is an important part of the phosphatidy‐lethanolamine (PE), which also consists of ethanolamine, phosphoric acid, and glycerol.^62^ PE as a major component of cell membrane phospholipids is important for maintaining the stability of cell structure. The precursors of PE, ethanolamine and ethanolamine phosphate, have been shown to inhibit the proliferation and metastasis of a variety of cancer cells.^63^ Significant tumor shrinkage was observed after 2 weeks of ethanolamine intervention in colon xenograft mice.^63^, ^64^ However, the gap in the specific biological mechanism of PE in CRC remains to be filled. For 2‐hydroxystearate, the study conducted by Hongping Xia et al. pointed out that 2‐hydroxystearate is highly expressed in hepatocellular carcinoma tissue with diabetes. This seems to imply that pyruvate can affect cancer through diabetes as a mediator.^65^

We also confirmed genetic predisposition to higher levels of gamma‐glutamylthreonine and 1,6‐anhydroglucose were detrimental to CRC. To date, there are no reports of gamma‐glutamylthreonine and CRC, with only one study indicating an increased risk of prostate cancer at high gamma‐glutamylthreonine concentrations.^66^ We attempted to speculate on the association of γ‐glutamine amino acids and CRC from the γ‐glutamine cycle due to γ‐glutamylcyclotransferase catalyzing the generation of various γ‐glutamyl amino acids from glutathione. γ‐glutamylcyclotransferase is highly expressed in a variety of cancers including CRC and is associated with poor prognosis in CRC.^67^, ^68^ High levels of γ‐glutamylcyclotransferase caused the accumulation of γ‐glutamine amino acids. This seems to imply an association between γ‐glutamine amino acids and the risk of CRC although further studies are needed to confirm this. For 1,6‐anhydroglucose, it is involved in glycolysis and pyruvate metabolism. 1,5‐anhydroglucitol, which has a similar structure to 1,6‐anhydroglucose, reflects blood glucose levels for1–2 weeks and is associated with type 1 diabetes while the reports on 1,6‐anhydroglucose are extremely limited.^69^ However, the specific effects of these metabolites on CRC need to be explored in detail under experimental conditions.

In addition, the high support of colocalization evidence from gene and protein wide was observed between LDHA and CRC. LDH catalyzes pyruvate to lactate, the last step of glycolysis. The elevation of LDH increases the rate of glycolysis, which in turn provides an energy source for cancer cells. Moreover, the acidic microenvironment resulting from the accumulation of lactate also promotes cancer cell invasion and angiogenesis.^44^ LDHA, a subtype of LDH, has been demonstrated to have excellent anticancer activities. Inhibition of LDHA enhances the sensitivity of cancer cells to chemotherapy, while suppressing cell proliferation, invasion, and epithelial‐to‐mesenchymal transition (EMT).^44^, ^70^ Transcription factors like c‐myc, HIF‐1, and p53 can inhibit LDHA activity, exerting anti‐tumor effects.^44^ Our colocalization analysis results also support that significant sites on GWAS signaling can affect CRC by influencing LDHA expression. Nevertheless, further experimental validation is needed to determine if LDHA can be a viable treatment target for CRC.

This MR analysis has several advantages. First and most critically this is the most complete and systematic study to date on exploring the causality between blood metabolites and CRC because in this work we analyzed 486 blood metabolites. Second, rigorous MR analysis was applied to reject the inevitable defects of previous studies such as reverse causality and confounding interference. In detail, to generate convincing estimates a series of methods are implemented to ensure that any factors that violate the MR assumptions are removed. The consistency of the three MR estimates in the direction and the sensitivity analysis demonstrated the robustness of the results. Third, the reliability of the results was further verified by additional GWAS data for replication analysis and meta‐analysis. Although the results of the replication analysis were not significantly attributable to differences in sample size, it showed consistent directionality with the primary analysis, which does not appear to have occurred by accident. Fourth, we assessed the heritability of IVs and the genetic correlation between metabolites and CRC using LDSC, which made the MR estimates more convincing. We also performed colocalization analysis to demonstrate, at both the gene and protein levels, that alterations in significant loci on GWAS signaling can influence CRC by regulating LDHA expression.

The current study also has several limitations. One limitation of our study is the limited number of SNPs available for the exposure of interest at the genome‐wide level. To address this, we set slightly relaxed thresholds for our MR analysis, a practice commonly adopted in other studies. However, the F‐statistic value for all selected SNPs exceeded 10, suggesting that our IVs were sufficiently robust. Moreover, the consistent causal direction supported by the results of the Steiger test lends credibility to our relaxed threshold setting. Second, to minimize the impact of ethnic variability, we only used GWAS data from individuals of European ancestry for this MR analysis. Hence, the generalizability of our findings to other populations warrants further exploration and validation. The third limitation of our study is that the precision of MR estimation partly relies on sample size. Therefore, expanding the sample size is necessary to confirm the reliability of our results. Additionally, while MR analysis provides valuable insights into etiology, it is important to note that our findings should be validated through rigorous RCTs and basic research before application in the clinic.

## 5 CONCLUSION

In summary, this MR study revealed that six genetically proxied blood metabolites have causal effects on CRC, and we identified nine metabolic pathways that could be implicated in the development of the CRC. LDHA deserve further investigation as potential therapeutic targets for CRC. The discovery of these serum metabolites provides valuable insights for early screening, prevention, and treatment of CRC, as well as for the design of future clinical studies. Moreover, this MR analysis combining genomics and metabolomics provides a reference direction for the exploration of the etiology and pathogenesis of CRC.

## AUTHOR CONTRIBUTIONS

**Zhangjun Yun:** Formal analysis (lead); methodology (lead); software (lead); validation (lead); writing – original draft (lead). **Ziwei Guo:** Resources (lead); software (lead); validation (lead); writing – original draft (lead). **Xiao Li:** Formal analysis (equal); validation (equal); visualization (equal). **Yang Shen:** Data curation (equal); investigation (equal). **Mengdie Nan:** Investigation (equal). **Qing Dong:** Conceptualization (equal); project administration (equal); supervision (equal); writing – review and editing (equal). **Li Hou:** Conceptualization (lead); funding acquisition (lead); methodology (lead); project administration (lead); supervision (lead); writing – review and editing (lead).

## FUNDING INFORMATION

This study was supported by the National Natural Science Foundation of China Grant Program (Grant/Award Number: 81573959), Capital Health Development Research Fund (Grant/Award Number: 2020–2‐4193 and 2022–1‐4171).

## CONFLICT OF INTEREST STATEMENT

The authors declare no conflicts of interest.

## ETHICS STATEMENT

All data in this study are available in publicly available databases. No additional ethical approval was required.

## Supporting information


**Figure S1.** Forest plots for the Mendelian randomization (MR) leave‐one‐out analysis of the significant inverse variance weighted (IVW) estimates.

**Table S1.** List of the identification (ID) for each of the 486 blood metabolites.
**Table S2**.
**Table S3**. Outliers identified by Radial MR.
**Table S4**. The results of MR‐PRESSO.
**Table S5**. The SNP‐based heritability (h2) of the metabolites.
**Table S6**. Estimation of the Steiger direction test from eight blood metabolites to colorectal cancer.
**Table S7**. The expression quantitative trait loci (pQTL) for blood LDHA.
**Table S8**. The protein quantitative trait loci (pQTL) for blood LDHA.
**Table S9**. Confounders identified from Phenoscanner.
**Table S10**. Metabolic pathways with significant enrichment of blood metabolites.

## Data Availability

We have annotated the article with the source of all original data, please contact the original authors for access if needed. The results of this study can be obtained by contacting the corresponding author.
