# Supplementary material for: Genetically predicted 486 blood metabolites in relation to risk of colorectal cancer: A Mendelian randomization study
Source: Cancer Med. 2023 May 3;12(12):13784–99. doi: 10.1002/cam4.6022 (PMC10315807; doi:10.1002/cam4.6022)
Supplement: Supplementary file 1 — Figure S1. Forest plots for the Mendelian randomization (MR) leave‐one‐out analysis of the significant inverse variance weighted (IVW) estimates. [file CAM4-12-13784-s001.docx]

**Supplementary Materials**

**Genetically predicted 486 blood metabolites in relation to risk**

**of colorectal cancer: A Mendelian randomization study**

**Zhangjun Yun Ziwei Guo Xiao Li Yang Shen Mengdie Nan Qing Dong**

**Li Hou**

**Supplementary Figure**

Supplementary Figure 1: Forest plots for the Mendelian randomization (MR) leave-one-out analysis of the significant inverse variance weighted (IVW) estimates.

**Supplementary Figure 1 Forest plots for the Mendelian randomization (MR) leave-one-out analysis of the significant inverse variance weighted (IVW) estimates.**

**
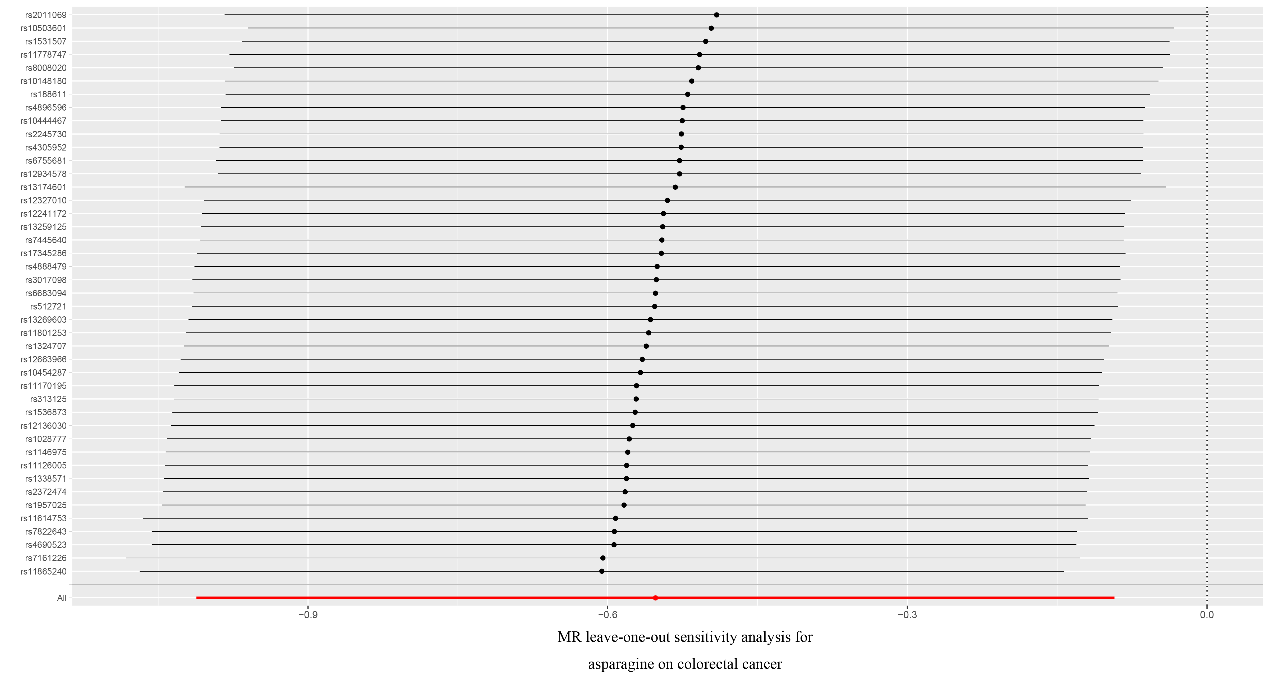

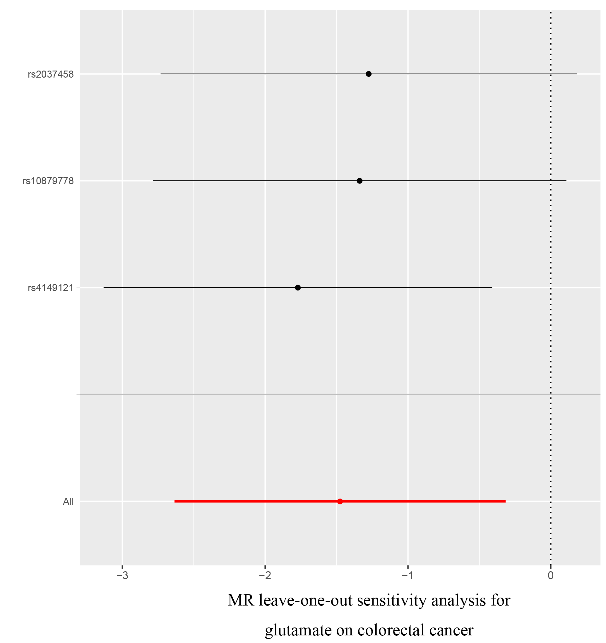

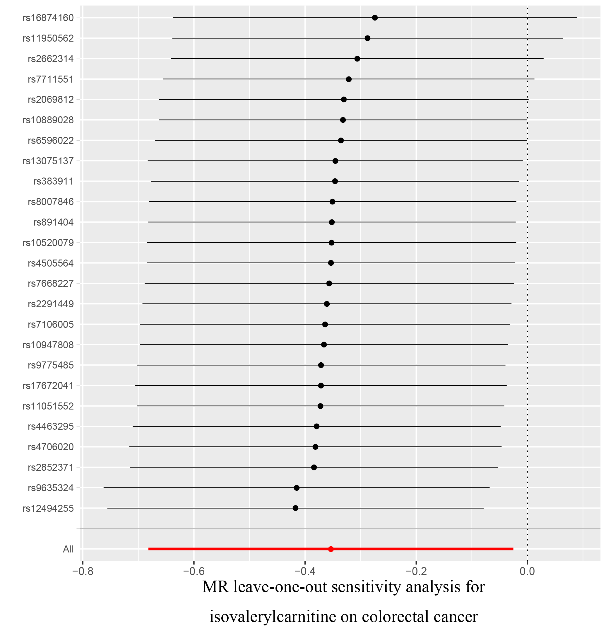
**

**
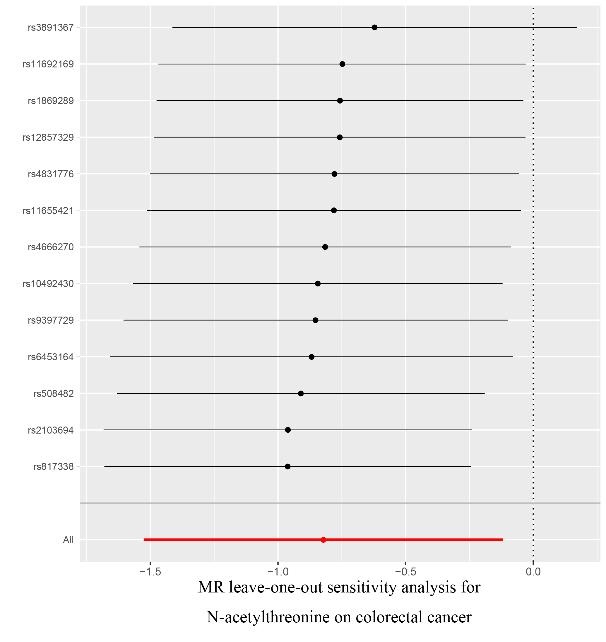

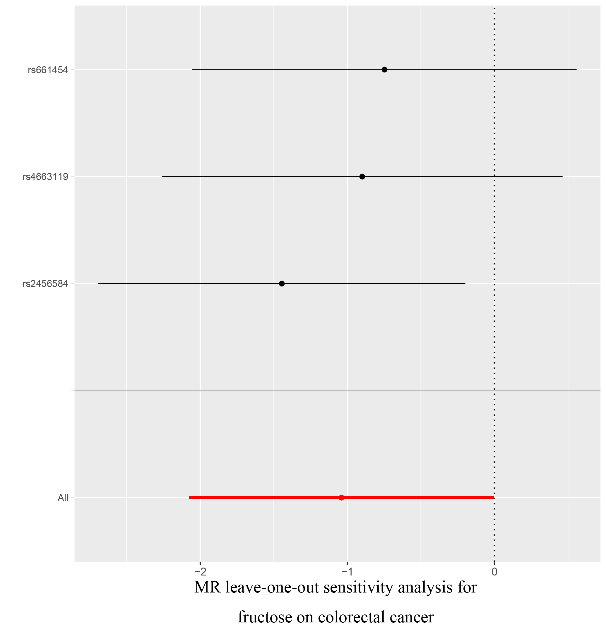
**

**
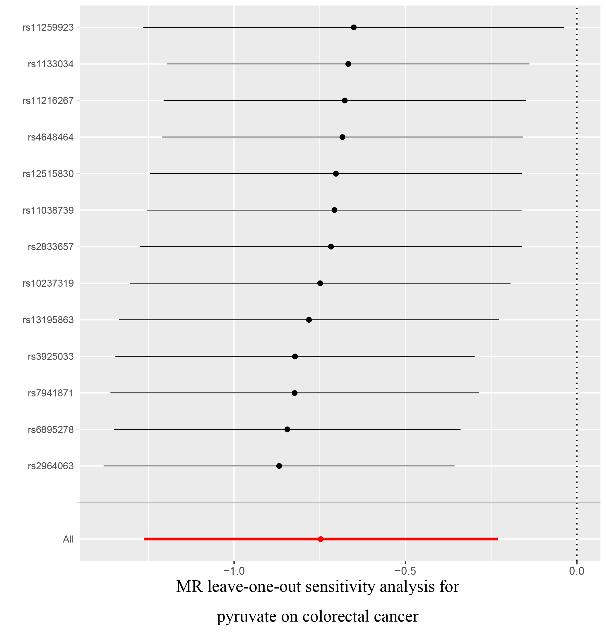

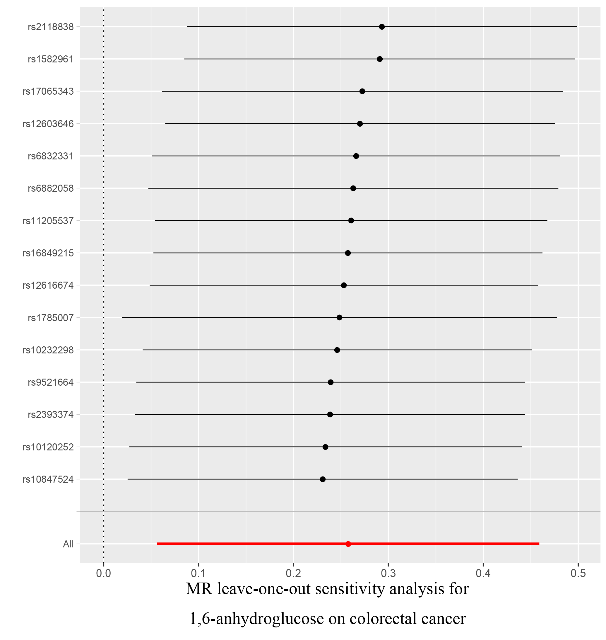
**

**
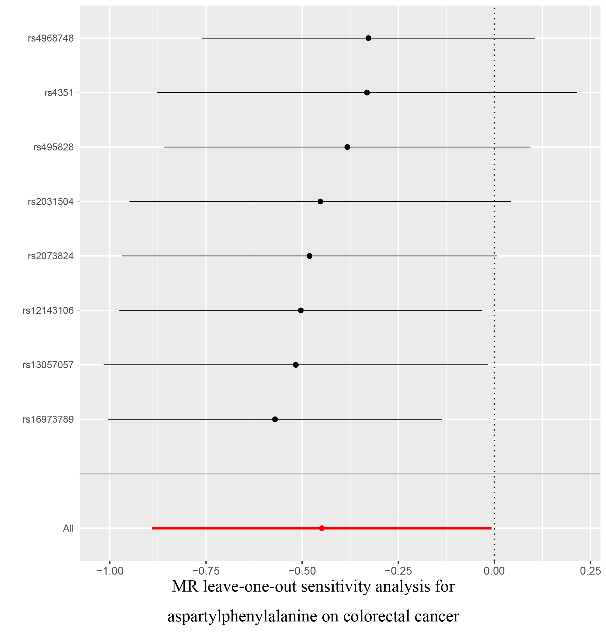

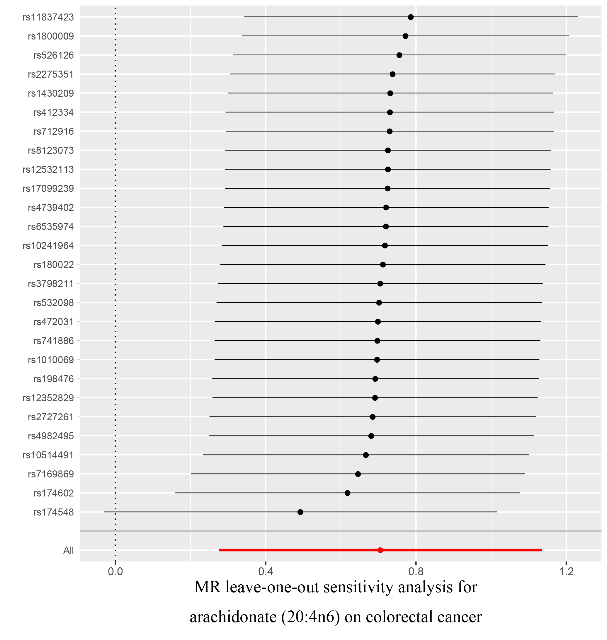
**

**
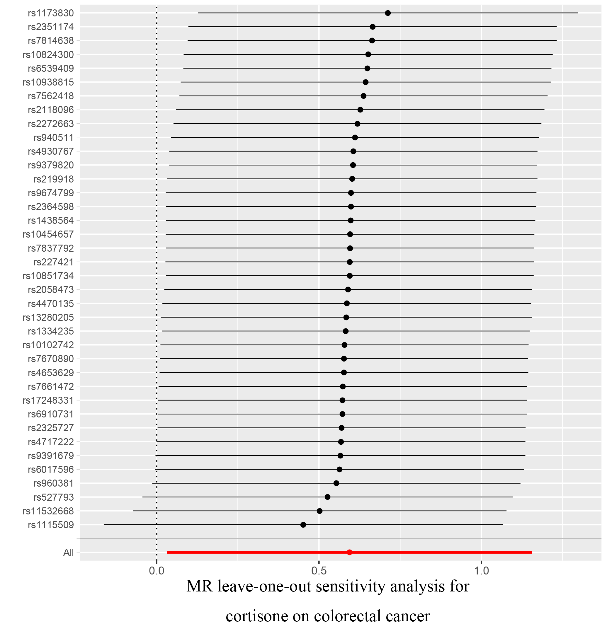

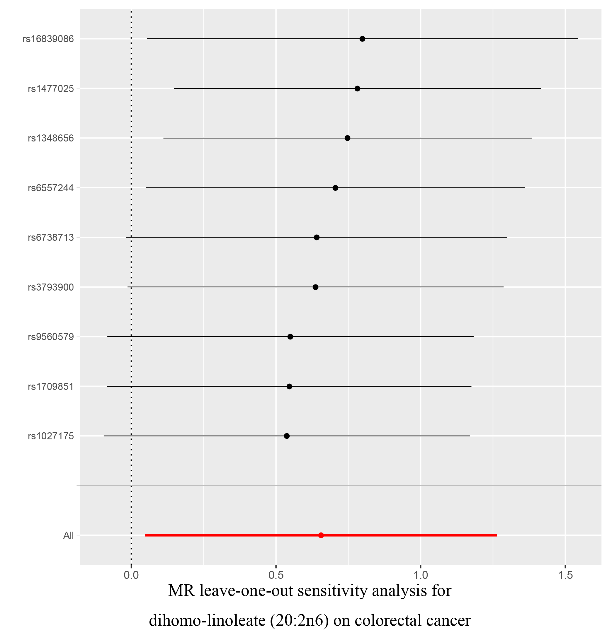
**

**
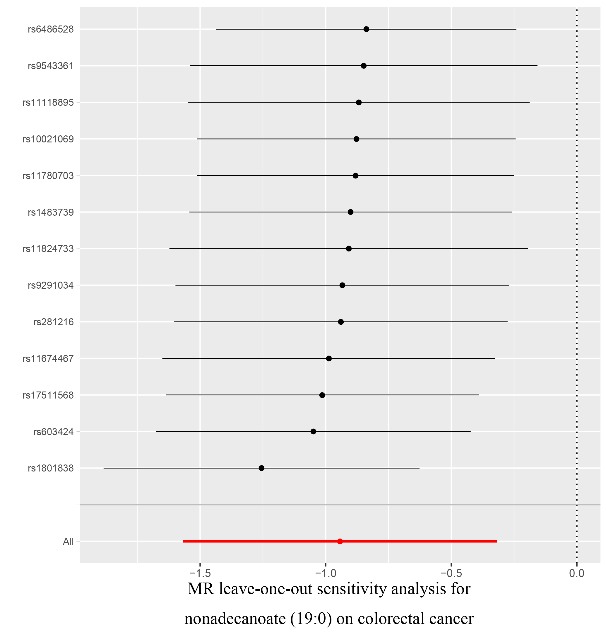

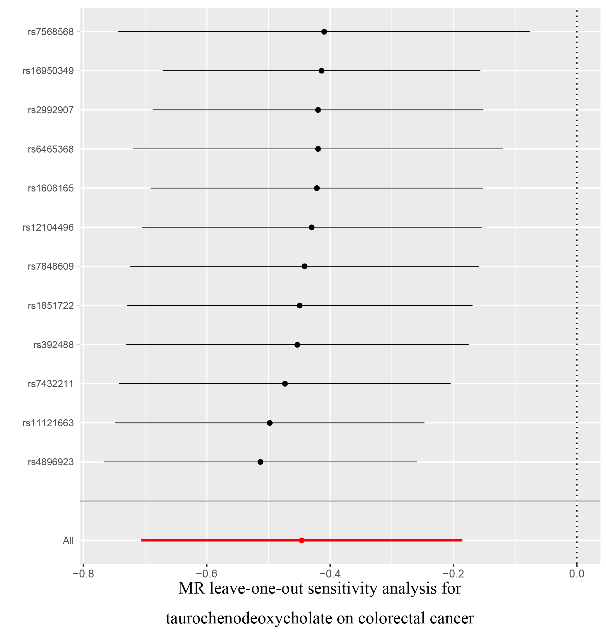
**

**
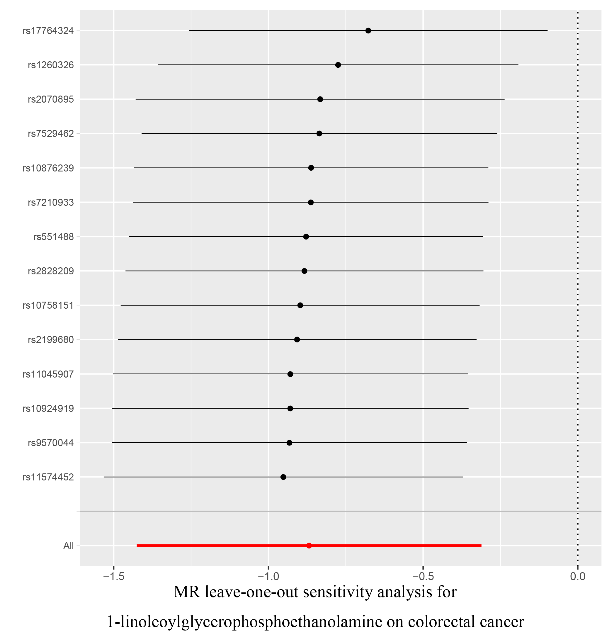

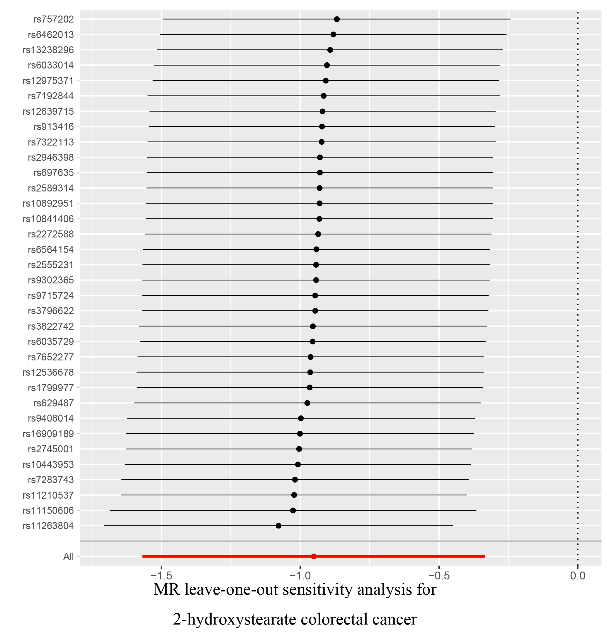
**

**
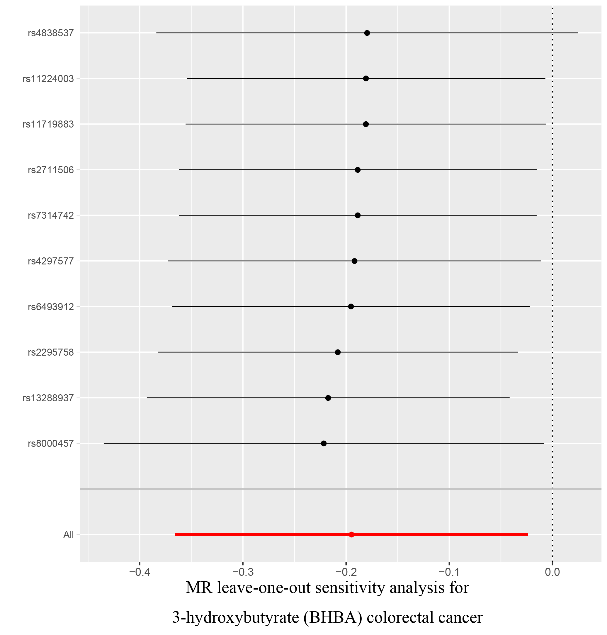

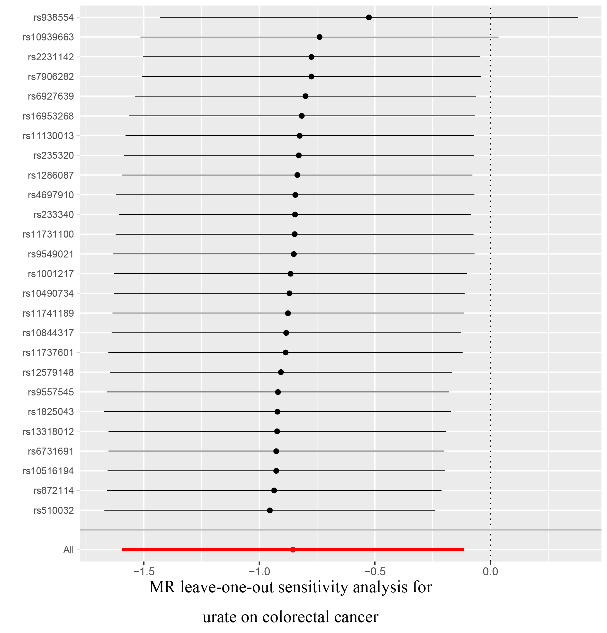
**

**
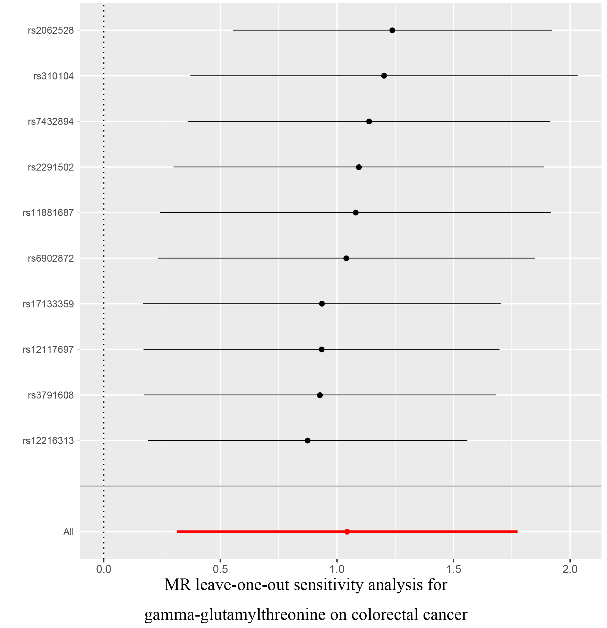

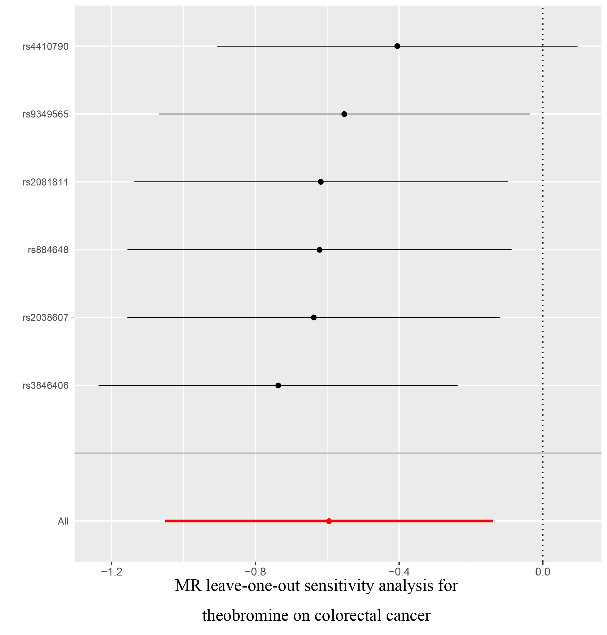
**

**
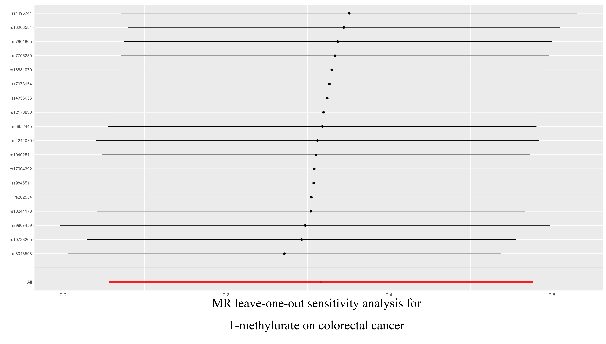

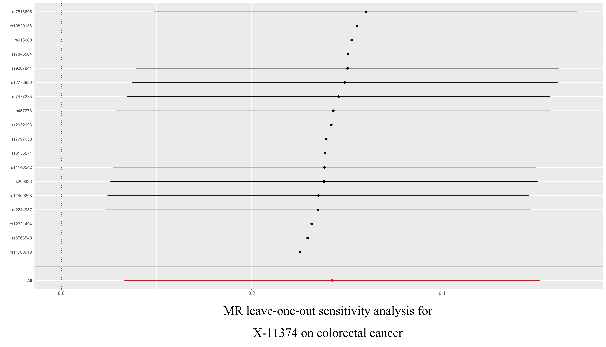
**

**
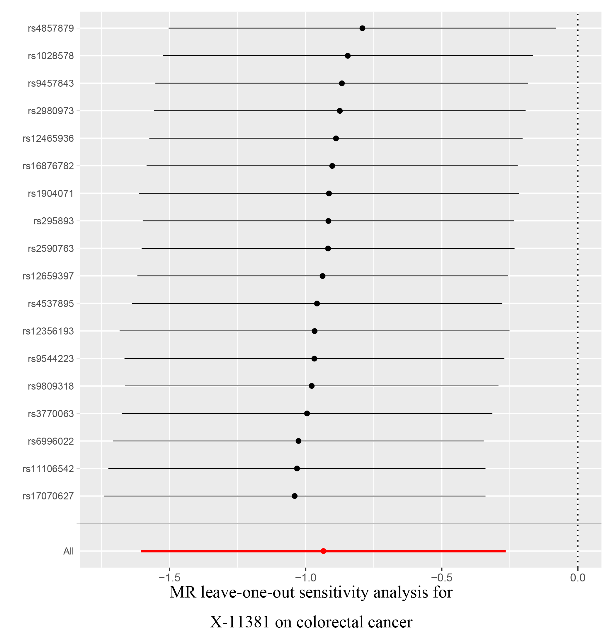

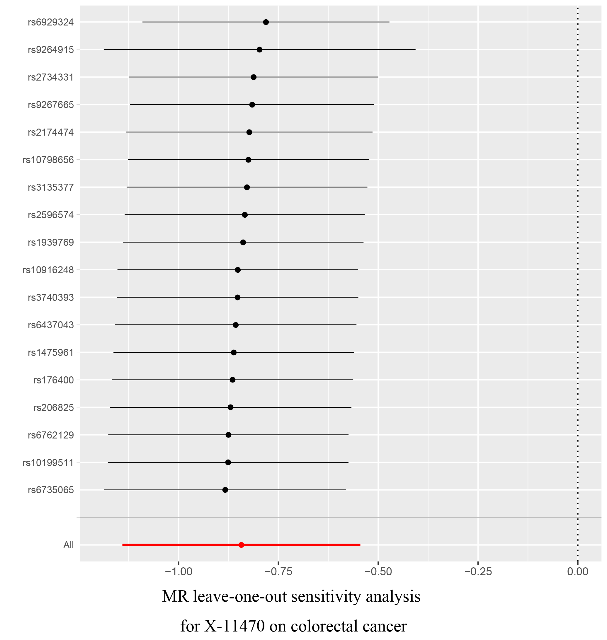
**

**
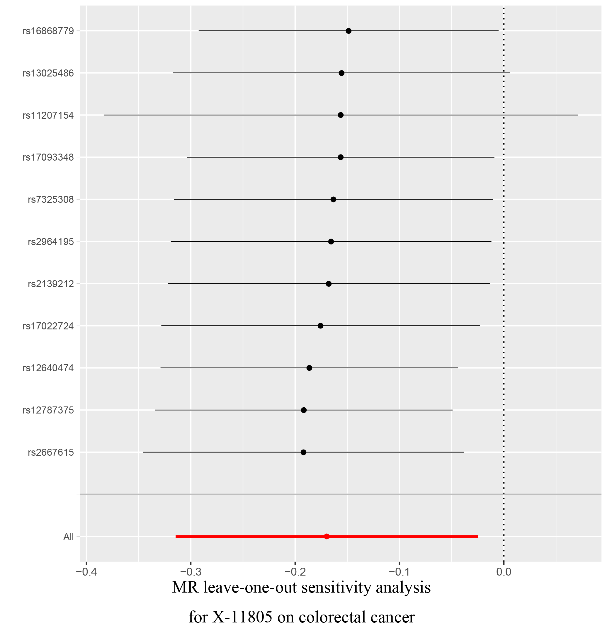

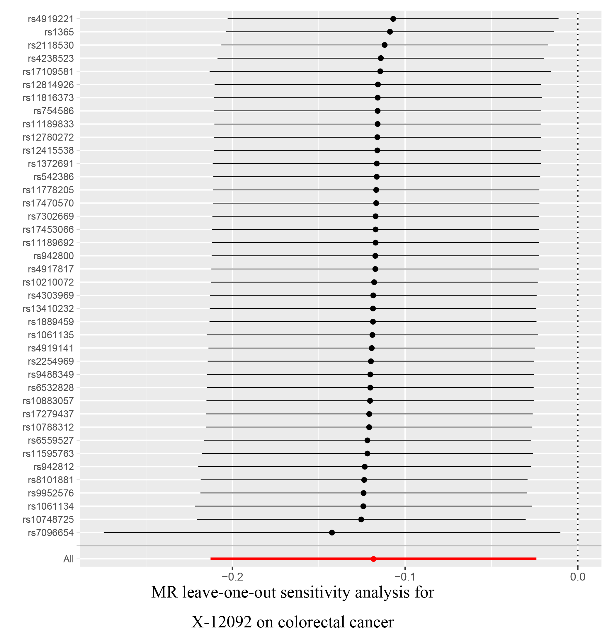
**

**
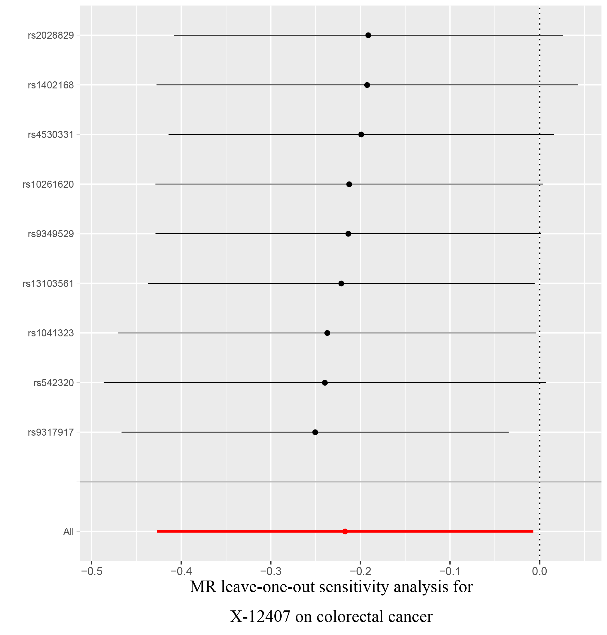

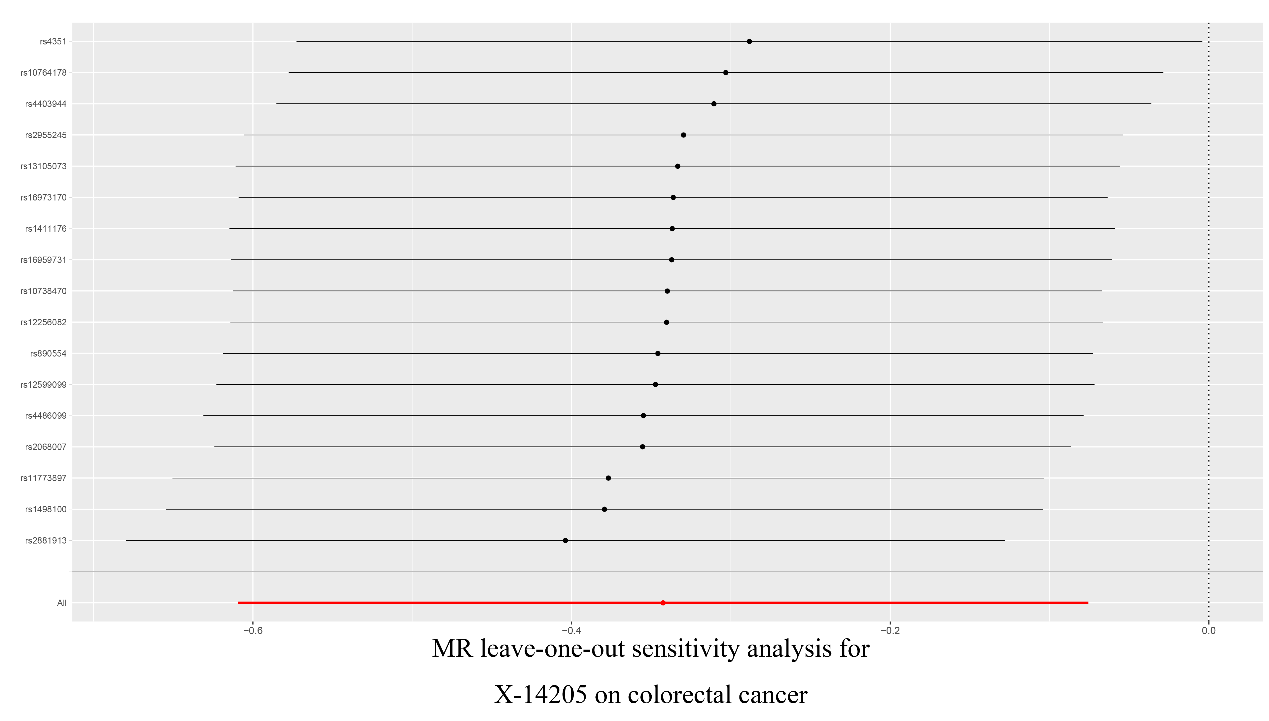
**
